# DUOX2-Driven Oxidative Stress Alters the Gut Redox Niche and Promotes Microbial Dysbiosis in Crohn’s Disease

**DOI:** 10.3390/antiox15030292

**Published:** 2026-02-26

**Authors:** Shu Xu, Xiaozhi Li, Xueting Wu, Kangrong Zheng, Youcai Yi, Yuqi Lin, Chunyang Tian, Yijun Zhu, Ce Tang, Shixian Hu, Shenghong Zhang, Yao He, Minhu Chen, Rui Feng

**Affiliations:** 1 Department of Gastroenterology, The First Affiliated Hospital, Sun Yat-sen University, Guangzhou 510080, China; 2 Institute of Precision Medicine, The First Affiliated Hospital, Sun Yat-sen University, Guangzhou 510080, China; 3 Department of Gastroenterology, Guangxi Hospital Division of the First Affiliated Hospital, Sun Yat-sen University, Nanning 530029, China

**Keywords:** Crohn’s disease, DUOX2, redox niche, microbiota fitness, epithelial-derived ROS

## Abstract

Crohn’s disease (CD) is characterized by chronic intestinal inflammation accompanied by gut dysbiosis and redox imbalance. We investigated the role of dual oxidase-2 (DUOX2), a major epithelial source of reactive oxygen species (ROS), in linking oxidative stress to microbe–host crosstalk. DUOX2 expression was upregulated in human intestinal samples and was positively associated with inflammatory readouts, oxidative stress indices, and dysbiosis. Intestinal epithelial cell-specific *Duox2* knockout (KO) mice exhibited reduced mucosal ROS, preserved barrier integrity, and attenuated dextran sodium sulfate (DSS)- and 2,4,6-trinitrobenzene sulfonic acid (TNBS)-induced colitis. Cohousing and fecal microbiota transplantation demonstrated that this protective phenotype was microbiota-dependent. Multi-omics profiling identified enrichment of *Parabacteroides*, particularly *P. distasonis*, in *Duox2* KO mice, and oral supplementation with *P. distasonis* enhanced resistance to colitis. Mechanistically, DUOX2-derived oxidative stress constrained *Parabacteroides* growth, as *P. distasonis* displayed marked susceptibility to hydrogen peroxide, with excessive intracellular ROS accumulation and an absence of key antioxidant defenses—including peroxide reductase C (AhpC) and superoxide dismutase B (SodB)—indicating that epithelial DUOX2 shapes a hostile luminal redox niche unfavorable to these beneficial microbes. Pharmacological inhibition of DUOX2 with Compound 521 reduced oxidative stress, ameliorated colitis, and partially restored microbial balance. These findings establish a DUOX2–ROS–microbiota axis in which epithelial DUOX2 amplifies oxidative stress, remodels the gut ecosystem, and promotes inflammation, and highlights DUOX2 suppression or ROS-sensitive *Parabacteroides* as potential redox-centric therapeutic strategies for CD.

## 1. Introduction

Crohn’s disease (CD) is a chronic, relapsing inflammatory bowel disease (IBD) characterized by segmental intestinal inflammation and progressive tissue damage [1]. Dysregulation of host–gut microbiota interactions is widely recognized as a central driver of CD pathogenesis [2]. Oxidative stress (OS), resulting from an imbalance between oxidants and antioxidant defenses, disrupts redox signaling and induces molecular damage [3]. Increasing evidence indicates that excessive OS impairs intestinal barrier integrity, alters mucin composition, and enhances bacterial translocation, collectively reshaping gut microbial ecology [4,5,6]. Despite its established role in CD, the upstream epithelial source of reactive oxygen species (ROS) and the downstream ecological consequences for gut microbes remain poorly understood.

In our prior work dissecting the OS-related determinants of CD, several intestinal OS-associated genes were identified that may modulate the development of CD by mediating host–microbiota interactions [7]. Through a large-scale intestinal transcriptome meta-analysis, dual oxidase 2 (DUOX2)—a member of the nicotinamide adenine dinucleotide phosphate (NADPH) oxidase family responsible for ROS production—was found to be among the most significantly upregulated CD-related OS genes, consistent with previous reports [8,9,10]. DUOX2 produces hydrogen peroxide (H_2_O_2_), and its chronic activation has been linked to redox imbalance, epithelial barrier disruption, and inflammation [11]. However, despite these associations, it remains unclear how DUOX2 alters gut microbial composition and whether DUOX2-derived ROS directly shape the luminal microbial ecosystem. Addressing these gaps is essential for determining whether DUOX2 functions as an inflammation-coupled redox amplifier in CD or serves as a modifiable redox node with therapeutic potential.

In this study, the associations among DUOX2 expression, inflammatory readouts, OS indices, and microbial dysbiosis were assessed in intestinal samples from patients with CD and experimental models. Using an intestinal epithelial cell (IEC)-specific *Duox2* knockout (KO) mouse model, our findings demonstrate that DUOX2 modulates intestinal inflammation through microbiota-dependent mechanisms. Among the gut microbial alterations, *Parabacteroides*, particularly *P. distasonis*, emerged as an ROS-sensitive taxon selectively constrained by DUOX2-derived OS. Mechanistically, DUOX2-dependent H_2_O_2_ accumulation constrained *P. distasonis* growth, as this species exhibited pronounced H_2_O_2_ sensitivity, excessive intracellular ROS accumulation, and a lack of key antioxidant enzymes, including peroxide reductase subunit C (AhpC) and superoxide dismutase B (SodB). These findings suggest that epithelial DUOX2-derived ROS establish a luminal redox microenvironment that is inhospitable to these potentially beneficial bacteria. Finally, pharmacological DUOX2 inhibition alleviated experimental murine colitis, reduced OS, and partially restored microbial balance, recapitulating phenotypes observed in *Duox2*-deficient mice. Collectively, this study aims to define the epithelial DUOX2-mediated orchestration of redox-dependent microbial ecology, identify antioxidant vulnerabilities in DUOX2-sensitive commensals, and highlight DUOX2 as a modifiable redox node with therapeutic relevance for CD.

## 2. Materials and Methods

### 2.1. Study Population, Specimen Collection, and Analysis

**Figure 1:**
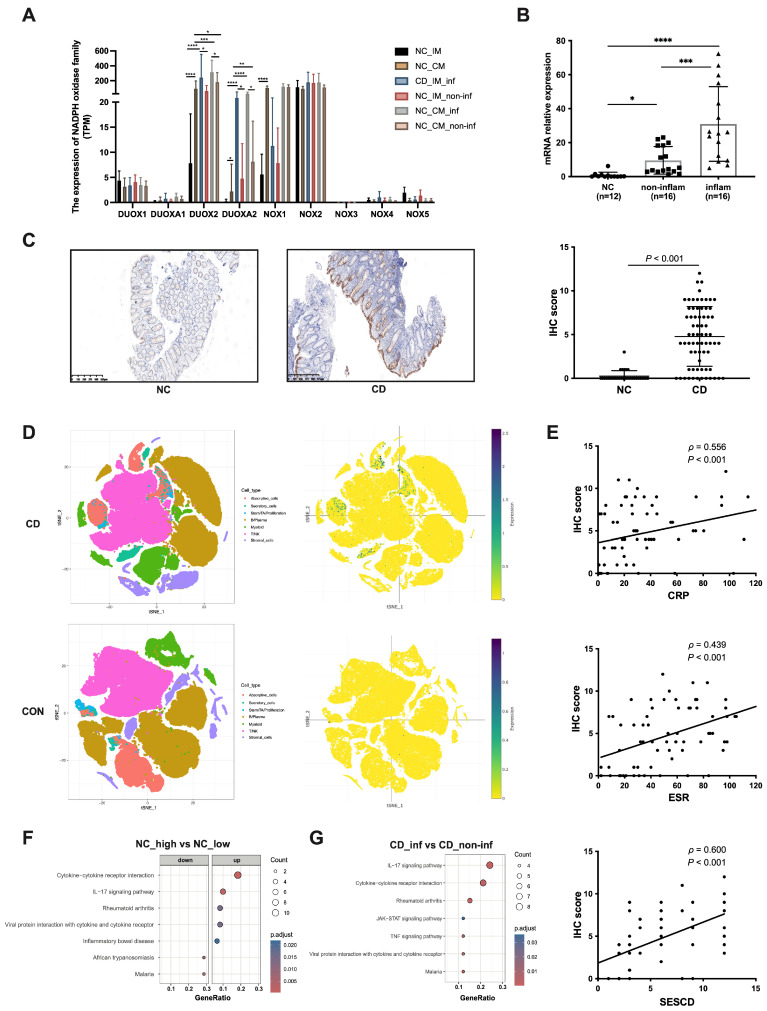
DUOX2 expression is upregulated and correlates with mucosal inflammation in patients with CD: (**A**) Expression profiles of the NADPH oxidase family members based on transcriptomic data from the FAH-SYS cohort. NC, normal control; IM, ileal mucosa; CM, colonic mucosa; inf, inflamed tissue; non-inf, non-inflamed tissue. (**B**) Relative expression levels of *DUOX2* in intestinal mucosal samples from three groups: patients with CD with non-inflamed mucosa (CD, non-inflam; *n* = 16), patients with CD with inflamed mucosa (CD, inflam; *n* = 16), and normal controls (NC; *n* = 12). (**C**) DUOX2 expression in endoscopic specimens from patients with CD (*n* = 69) and NC (*n* = 32) detected by immunohistochemical (IHC) staining. Left, representative IHC images; right, quantitative analysis of IHC scores. (**D**) Cellular localization of *DUOX2* based on single-cell RNA-Seq of colonic biopsies from patients with CD (*n* = 14) and controls (CON; *n* = 13) obtained from the Single Cell Portal. Left, uniform manifold approximation and projection (UMAP) plot; right, DUOX2 expression across single colonic populations. (**E**) Correlation analysis between DUOX2 expression and SES-CD, CRP, and ESR in patients with CD (*n* = 69). (**F**) KEGG pathway analysis of RNA-Seq data from normal control intestinal mucosa with high *DUOX2* expression and low *DUOX2* expression. Color intensity represents the *p.adjust* value (Benjamini–Hochberg multiple-testing correction), with *p.adjust* < 0.05 considered significantly enriched. (**G**) KEGG pathway analysis comparing RNA-Seq data from inflamed and non-inflamed intestinal regions in patients with CD; all pathways are upregulated (*p.adjust* < 0.05). Note: * *p* < 0.05, ** *p* < 0.01, *** *p* < 0.001, **** *p* < 0.0001.

A total of 46 treatment-naïve patients with CD and 44 normal controls (NCs) were prospectively recruited from the First Affiliated Hospital of Sun Yat-sen University (FAH–SYS) IBD cohort. Specimens from these participants were used for RNA sequencing (RNA-Seq) analysis (Figure 1A,F,G). Paired terminal ileal and colonic tissue samples were collected from CD patients (inflamed and non-inflamed regions) and NCs. Differential gene expression analysis was performed using the R package DESeq2 (version 1.42.1), with significance thresholds set at *p* < 0.05 and |log_2_ fold change| > 1. Kyoto Encyclopedia of Genes and Genomes (KEGG) pathway enrichment analysis was conducted using the R package clusterProfiler (v4.10.1).

A validation cohort consisting of 69 patients diagnosed with CD and 32 NCs was enrolled, with human intestinal tissues collected from all participants. The clinical characteristics and disease activity data of the cohort are summarized in Table S1. These samples were used to obtain the data shown in Figure 1B,C,E. Disease activity was assessed using the Simple Endoscopic Score for Crohn’s Disease (SES-CD) [12], serum C-reactive protein (CRP), and erythrocyte sedimentation rate (ESR).

A large paired dataset of intestinal RNA-Seq data and mucosal 16S rRNA sequencing data from patients with CD was obtained from a Dutch cohort (*n* = 679) [13]. Alpha diversity was calculated using the Shannon and Chao1 indices at the genus level. Group differences were assessed using the Mann–Whitney U test. Beta diversity was calculated using Bray–Curtis distance, and the proportion of explained variance (R^2^) was assessed via permutational multivariate analysis of variance (PERMANOVA) using the adonis function in the vegan R package v2.6.6.1. A dysbiosis index for each participant was calculated as the median Euclidean distance between their gut microbial composition (genus level) and a reference population; for patients with CD, this represented the distance to all normal controls, with higher values indicating more severe dysbiosis [14]. Linear regression models were employed to identify genes whose expression levels were significantly associated with dysbiosis scores (false discovery rate, FDR < 0.05). An extreme gradient boosting (XGBoost) model was used to predict dysbiosis scores from gene expression data, and SHapley Additive exPlanations (SHAP) values were calculated using SHAPforxgboost (v0.1.3) to quantify feature importance and directionality. Differential microbial features were identified using linear discriminant analysis effect size (LEfSe).

Single-cell RNA sequencing (scRNA-Seq) data for analyzing DUOX2 expression, including 14 CD samples and 13 control samples, were retrieved from the public database Single Cell Portal [15]. Cell annotations were obtained from two studies (PREDICT 2021 paper: CD; and PREDICT 2021 paper: FGID), and corresponding cell types were merged for analysis.

### 2.2. Mouse Strains

Dextran sodium sulfate (DSS)- and 2,4,6-trinitrobenzene sulfonic acid (TNBS)-induced colitis models were established using C57BL/6J and BALB/c mice, respectively (GemPharmatech Co., Ltd., Nanjing, China). Villin-Cre mice were obtained from Jackson Laboratory (Bar Harbor, ME, USA), and *Duox2* floxed mice on a C57BL/6J background were generated by GemPharmatech Co., Ltd. Mouse genotypes were confirmed via DNA amplification before model construction.

### 2.3. DSS and TNBS Treatment

For DSS-induced colitis, mice were administered with 2% DSS (MP Biomedicals, Irvine, CA, USA) with a molecular weight (MW) of 36,000–50,000 Da in drinking water ad libitum for 7 days, according to the established protocol [16]. After DSS solution withdrawal, the disease activity index (DAI) was measured as previously described [16]. Histological scoring (grades 0–3) was performed on conventional paraffin-embedded, hematoxylin and eosin (H&E)-stained sections by two independent investigators [16].

TNBS (Sigma-Aldrich, St. Louis, MO, USA)-induced colitis was generated as previously described [16]. Mice were anesthetized with isoflurane, and 120 μL of 2.5% (*w*/*v*) TNBS in 50% absolute ethanol was administered intrarectally 7 days after pre-sensitization with 150 μL of 1% (*w*/*v*) TNBS. Mice were euthanized via cervical dislocation at 3–7 days post-enema, and samples were collected during the active disease phase. Histological assessment was conducted on harvested tissues.

### 2.4. Quantitative Reverse Transcription–Polymerase Chain Reaction (qRT-PCR)

For human and mouse specimens, total RNA was extracted from cultured cells or tissue biopsies using TRIzol Reagent (Invitrogen, Carlsbad, CA, USA), following the manufacturer’s instructions. RNA concentration and purity were quantified using a NanoDrop 2000 spectrophotometer (Invitrogen, USA). Reverse transcription of RNA to cDNA was performed in vitro using the Transcriptor First Strand cDNA Synthesis Kit (Roche, Basel, BS, Switzerland), and qRT-PCR was conducted with FastStart Universal SYBR Green Master Mix (Roche, Switzerland). Relative gene expression levels were calculated using the delta-delta Ct (ΔΔCt) method, with β-actin as the internal reference gene. All primer sequences used for this assay are listed in Table S2.

For quantification of bacterial abundance analysis in mouse feces, total DNA was extracted using a DNA Extraction Kit (Magen, Guangzhou, China) according to the manufacturer’s instructions. The relative abundance of target bacterial taxa was measured via qPCR, using the 16S rRNA gene as the internal reference. Primer sequences for these analyses are also listed in Table S2.

### 2.5. Western Blotting

Tissue samples were lysed in RIPA buffer (Cell Signaling Technology, Danvers, MA, USA) supplemented with a protease and phosphatase inhibitor cocktail (Invitrogen, USA). SDS-polyacrylamide gel electrophoresis (SDS-PAGE) was performed as previously described [17]. After electrophoresis, proteins were transferred onto membranes, which were subsequently blocked with skim milk for 1 h at room temperature and then incubated with primary antibodies at 4 °C overnight (anti-DUOX2 antibody, Abcam, Cambridge, UK). Membranes were then incubated with appropriate secondary antibodies for 1 h at room temperature. Immunoblotting signals were visualized with horseradish peroxidase (HRP) substrate (Millipore, Darmstadt, Hessen, Germany) and captured with an iBright FL1500 imaging system (Invitrogen, USA). Tubulin served as the internal reference for normalization. Semi-quantitative band density analysis was conducted using the Image-Pro Plus software (v6.0), and the results are presented as bar charts.

### 2.6. Immunohistochemistry (IHC) Assay

Tissue sections were prepared from paraffin-embedded human and mouse tissues. After deparaffinization and gradient hydration, the sections were subjected to antigen retrieval; subsequently, they were incubated with 3% hydrogen peroxide for 15 min to expose epitopes and quench endogenous peroxidase activity. Sections were blocked with 3% bovine serum albumin (BSA) in phosphate-buffered saline (PBS) for 1 h at room temperature, then incubated with the primary antibody overnight at 4 °C (anti-DUOX2 for human: 1:400 dilution; anti-DUOX2 for mouse: 1:200 dilution, Abcam, UK). After three washes with PBS containing 0.1% Tween-20, sections were incubated with HRP-conjugated secondary antibodies (Cell Signaling Technology, USA) for 30 min at room temperature. Following counterstaining with hematoxylin, serial ethanol dehydration, and coverslip mounting, images were captured using a Leica DMI1 microscope (Leica Microsystems, Wetzlar, Germany).

### 2.7. In Vivo Intestinal Permeability Assessment

Fluorescein isothiocyanate-dextran (FD4; MW 4000 Da; Sigma-Aldrich, St. Louis, MO, USA) was administered via oral gavage at a dosage of 22 mg·kg^−1^ after the mice were fasted for 4 h. Blood was collected via orbital extirpation and centrifuged at 1900× *g* for 10 min. The supernatant was further centrifuged again at 16,000× *g* for 10 min. Plasma samples and gradient-diluted FD4 standards were measured using a Spectra Max M5 microplate reader (Molecular Devices, San Jose, CA, USA) at excitation/emission wavelengths of 490/530 nm.

### 2.8. Oxidative Stress Index Assays

H_2_O_2_ levels in intestinal epithelial cells were quantified using the Amplex Red Assay Kit (Thermo Fisher Scientific, Waltham, MA, USA), following the manufacturer’s instructions. Fluorescence intensity was measured with a multi-mode microplate reader (excitation 530–560 nm; emission 590 nm).

ROS levels were assessed using sample-type-specific methods. For tissue sections, dihydroethidium (DHE) staining was performed. Frozen sections were thawed, incubated with DHE solution (1:500, Servicebio, Wuhan, China) in the dark for 20–30 min at 37 °C, rinsed with PBS, and visualized using an Olympus BX-63 microscope (Olympus, Tokyo, Japan). ROS levels were semi-quantified by measuring the mean fluorescence intensity using the ImageJ software (v1.52a). For cultured cells and microbes, ROS levels were measured using 2′,7′-dichlorodihydrofluorescein diacetate (DCFH-DA) (MCE, Shanghai, China) in accordance with standard protocols.

Superoxide dismutase (SOD) levels in specimens were measured using an SOD Assay Kit (Beyotime Biotechnology, Shanghai, China). Assays were performed according to the manufacturer’s instructions, and SOD activity was calculated from absorbance readings at 560 nm.

### 2.9. Cell Culture and Adenovirus Transfection

NCM460 cells, a human normal colonic mucosal epithelial cell line (INCELL, San Antonio, TX, USA), were cultured in M3: BaseF medium (INCELL, USA) supplemented with 10% fetal bovine serum and 1× penicillin–streptomycin (Invitrogen, USA) in a 5% CO_2_ atmosphere at 37 °C.

Adenovirus for DUOX2 overexpression and negative control constructs were designed and generated by Genecopoeia Inc. (Shanghai, China). Cells were transfected with adenovirus according to the manufacturer’s instructions using a pre-optimized multiplicity of infection (MOI). Fresh complete medium was replaced at 12–24 h post-transfection. Transfection efficiency was evaluated using fluorescence microscopy at 24–48 h post-transfection. Transient DUOX2 overexpression lasted for approximately 7 days.

### 2.10. Cohousing Experiment

Age- and sex-matched wild-type (WT) and *Duox2* KO mice from the same breeders were assigned to either separate housing or cohousing for 3–4 weeks. After this period, all mice were subjected to DSS-induced colitis modeling.

### 2.11. Antibiotic Cocktail (ABX) Experiment

Age- and sex-matched WT and *Duox2* KO mice from the same breeders were assigned to separate housing and administered a six-antibiotic cocktail (1000 mg kanamycin, 87.5 mg gentamicin, 142.5 mg colistin, 537.5 mg metronidazole, 112.5 mg vancomycin, and 25 mg erythromycin dissolved in 2.5 L distilled water) for 14 days to deplete their gut microbiota. All antibiotics were purchased from Maclin Inc. (Shanghai, China).

### 2.12. Fecal Microbiota Transplantation (FMT) Experiment

Age- and sex-matched WT and *Duox2* KO mice assigned to separate housing were first treated with the six-antibiotic cocktail described above to deplete their gut microbiota. Each mouse was administered 200 μL of a PBS suspension containing feces from either WT or DUOX2 KO donor mice via oral gavage. Three days after FMT, all mice underwent DSS-induced colitis modeling.

### 2.13. Microbial Strains and Culturing Conditions

Two strains of *Parabacteroides distasonis* (ATCC BAA-1295, ATCC 8503) were purchased from the Guangdong Microbial Culture Collection Center (Guangzhou, China) and cultured anaerobically at 37 °C in BHC medium. *Escherichia coli* strain MG1655 was cultured under the same anaerobic conditions.

To establish a bacterial OS model, BHC medium was supplemented with H_2_O_2_ at final concentrations of 0.2, 0.5, and 1.0 mM. After 24 h of anaerobic incubation at 37 °C, bacterial growth was monitored by OD_600_ values.

### 2.14. Fecal DNA Extraction and 16S rRNA Sequencing

Fecal samples were collected from *Duox2* KO mice and their WT littermates. DNA was extracted using the FastPure Stool DNA Isolation Kit (MJYH, Beijing, China) according to the manufacturer’s instructions. The V3–V4 hypervariable regions of the bacterial 16S rRNA gene were amplified by PCR using bacterial universal primers 341F (5′–CCTACGGGNGGCWGCAG–3′) and 806 R (5′–GGACTACHVGGGTATCTAAT–3′). Amplicons were excised from 2% agarose gels, purified with the AxyPrep DNA Gel Extraction Kit (Axygen Biosciences, Union City, CA, USA), and quantified using an ABI StepOnePlus Real-Time PCR System (Life Technologies, Carlsbad, CA, USA). Purified amplicons were pooled in equimolar amounts and paired-end sequenced (PE250) on an Illumina platform according to standard protocols.

Raw sequences were demultiplexed, quality filtered with fastp (v0.19.6) [18], and merged using FLASH (v1.2.11) [19]. High-quality sequences were denoised using DADA2 [20] in QIIME2 (v2020.2) [21] to generate amplicon sequence variants (ASVs). Sequencing depth was normalized by rarefying samples to 20,000 sequences per sample, achieving an average Good’s coverage of 97.90%. Taxonomic assignment was performed in QIIME2 using a naïve Bayes classifier against the SILVA 16S rRNA database. Bioinformatics analysis of the gut microbiota was carried out using the Majorbio Cloud platform, and alpha diversity, beta diversity, and LEfSe analyses were performed as described previously.

### 2.15. Shotgun Metagenomic Sequencing

Fecal DNA quality, concentration, and purity were assessed using a SynergyHTX, NanoDrop2000, and 1% agarose gel electrophoresis. DNA was fragmented to ~350 bp using the Covaris M220 (Gene Company Limited, Hong Kong, China), libraries were constructed with the NEXTFLEX Rapid DNA-Seq Kit, and sequencing was performed on an Illumina NovaSeq™ X Plus using the NovaSeq X Series 25B Reagent Kit (Majorbio, Shanghai, China).

Raw metagenomic reads were processed on the Majorbio Cloud Platform. Adapter trimming and quality control were performed using fastp (v0.23.0), and host DNA was removed using BWA [22] (v0.7.17). Clean reads were assembled with MEGAHIT (v1.1.2) [23], retaining contigs ≥ 300 bp. Open reading frames (ORFs) were predicted using Prodigal (v2.6.3) [24] and clustered into a non-redundant gene catalog using CD-HIT (90% sequence identity, 90% coverage) [25]. Gene abundance was estimated using SOAPaligner at 95% identity [26].

For functional profiling of OS, high-quality reads filtered by KneadData v0.12.0 were aligned against the SEED database using translated homology search and annotated to subsystems and functional levels 1–3 using Super-Focus [27].

### 2.16. Single-Cell RNA Sequencing

Two *Duox2* KO mice and two WT mice were used. Isolation of murine intestinal epithelial cells was performed as described previously [28]. Single-cell barcoding was performed using a Chromium Single-Cell Controller (10× Genomics, Pleasanton, CA, USA), and reverse transcription was carried out using an S1000 Touch Thermal Cycler (Bio-Rad, Hercules, CA, USA). Libraries were prepared and sequenced on an Illumina NovaSeq 6000 sequencer (Illumina, San Diego, CA, USA). After read processing and quality control, cells were clustered using Seurat v3.1 with SCTransform normalization, and cell clusters were visualized via uniform manifold approximation and projection (UMAP) plots. Functional enrichment analysis was performed using Gene Ontology (GO) analysis.

### 2.17. Protein Extraction and Astral DIA Proteomics Sequencing

Three *Parabacteroides distasonis ATCC BAA-1295* (PD1) and three *Escherichia coli MG1655* (EC) samples were prepared for proteomic sequencing analysis. Cultures were grown to the logarithmic phase, centrifuged at 5000× *g* for 10 min at 4 °C, and the pellets were washed twice with pre-cooled sterile PBS and stored at −80 °C for further use.

Proteins were extracted using lysis buffer and quantified with using a BCA Protein Assay Kit (P0012, Biocentury, Shanghai, China). The quality of the extracted proteins was evaluated through SDS-PAGE and Coomassie Brilliant Blue R-250 staining. Mixed samples containing 15 μg of protein from each sample were prepared for database construction and quality control. All samples underwent trypsin digestion via filter-aided sample preparation [29], were desalted with C18 cartridges, and the resulting peptides were lyophilized and reconstituted in 0.1% formic acid. Each sample was supplemented with indexed retention time (iRT) peptides, which were added for data-independent acquisition (DIA) calibration. Peptides were analyzed using a nanoscale Vanquish Neo UHPLC system (Thermo Scientific, Waltham, MA, USA) in DIA mode coupled with an Astral high-resolution mass spectrometer (Thermo Scientific, USA). In the first stage of mass spectrometry (MS), precursor ions were scanned over a mass range spanning 380 to 980 *m*/*z* at a resolution of 240,000 (200 *m*/*z*), with a normalized automatic gain control (AGC) target of 500% and an injection time of 5 ms. In the second stage of MS, the DIA scans utilized 300 scanning windows (2 *m*/*z* isolation width), and higher-energy collisional dissociation was applied at 25 eV, a normalized AGC target of 500%, and a maximum injection time of 3 ms.

DIA-NN (v1.9.2) was used for data analysis with trypsin digestion (maximum missed cleavage of 1), carbamidomethyl^©^ as a fixed modification, and oxidation (M) and N-terminal acetylation as variable modifications. Proteins were reported at a 99% confidence level and an FDR of ≤0.01, confirmed via the UniProt database.

Data were analyzed using Personalbio Genescloud (www.genescloud.cn). Principal component analysis (PCA) was used to visualize group differences. Differential analysis between two groups was performed using a *t*-test with significance thresholds of *p* < 0.05 and fold change of FC ≥2 or ≤0.5. Functional enrichment analysis was conducted using the R package clusterProfiler (v4.10.0).

### 2.18. Statistical Analysis

Data were analyzed using the GraphPad Prism 9.0 software (GraphPad Software Inc., San Diego, CA, USA) and IBM SPSS Statistics 23 (IBM Corp., Armonk, NY, USA). Values are presented as the mean ± standard error of the mean (SEM). For normally distributed data, group comparisons were performed using an unpaired, two-tailed Student’s *t*-test or one-way analysis of variance (ANOVA). Non-normally distributed data were analyzed with the Mann–Whitney U-test and Kruskal–Wallis test. Spearman correlation was calculated to evaluate the relationship between DUOX2 expression and disease activity. *p* < 0.05 was considered statistically significant.

## 3. Results

### 3.1. DUOX2 Expression Is Markedly Upregulated and Closely Associated with Mucosal Inflammation in Patients with CD

To characterize epithelial OS-related gene expression in CD, transcriptomic profiles of the NADPH oxidase family were analyzed across healthy, non-inflamed, and inflamed intestinal tissues using RNA-Seq data from an independent FAH-SYS cohort (Figure 1A, Table S3). In normal intestinal mucosa, *DUOX2*, dual oxidase maturation factor 2 (*DUOXA2*), and NADPH oxidase 1 (*NOX1*) exhibited higher expression in the colonic mucosa than in the ileal mucosa, whereas NADPH oxidase 2 (*NOX2*) showed relatively high expression levels in both regions. Under inflammatory conditions, however, only *DUOX2* and its maturation factor *DUOXA2* showed marked inflammation-dependent induction, with significantly higher expression in inflamed compared with non-inflamed ileal and colonic mucosa. These results identify DUOX2 as the most inflammation-responsive NADPH oxidase in CD.

Elevated DUOX2 expression in patients with CD was subsequently confirmed in independent patient cohorts. Quantitative PCR confirmed significantly increased *DUOX2* mRNA levels in inflamed mucosa compared with non-inflamed tissues and normal controls (Figure 1B). Consistently, IHC analysis revealed increased DUOX2 protein abundance predominantly localized to epithelial cells (Figure 1C), a pattern further corroborated by publicly available scRNA-Seq datasets (Figure 1D). Clinically, DUOX2 expression showed significant positive correlations with indices of disease severity, including endoscopic SES-CD score, serum CRP, and ESR (Figure 1E), indicating a close association among DUOX2 levels, mucosal inflammatory activity, and systemic inflammatory burden.

To determine whether DUOX2 induction represents a conserved inflammatory feature, we evaluated DUOX2 expression in DSS- and TNBS-induced colitis models. DUOX2 abundance was substantially elevated in both models compared with their corresponding control groups (Figure S1A,B). In the DSS model, DUOX2 expression increased progressively from early inflammation (day 4) to the acute phase (day 9) and declined during the recovery phase (day 20), closely paralleling the dynamic inflammation-dependent expression pattern observed in CD mucosa (Figure S1C,D).

To delineate transcriptional pathways associated with *DUOX2* activation, normal control mucosal samples were stratified based on *DUOX2* expression levels, followed by differential gene expression and pathway enrichment analyses. Compared with low-*DUOX2* tissues, high-*DUOX2* mucosa showed robust upregulation of inflammatory signaling pathways, including interleukin 17 (IL-17) signaling, tumor necrosis factor (TNF) signaling, neutrophil activation, and canonical IBD pathways (Figure 1F). Notably, these *DUOX2*-associated pathways substantially overlapped with those enriched in inflamed relative to paired non-inflamed CD mucosa (Figure 1G), indicating that *DUOX2* upregulation is embedded within a broad inflammatory transcriptional program.

### 3.2. DUOX2 Upregulation Is Associated with Gut Microbial Dysbiosis in CD

**Figure 2:**
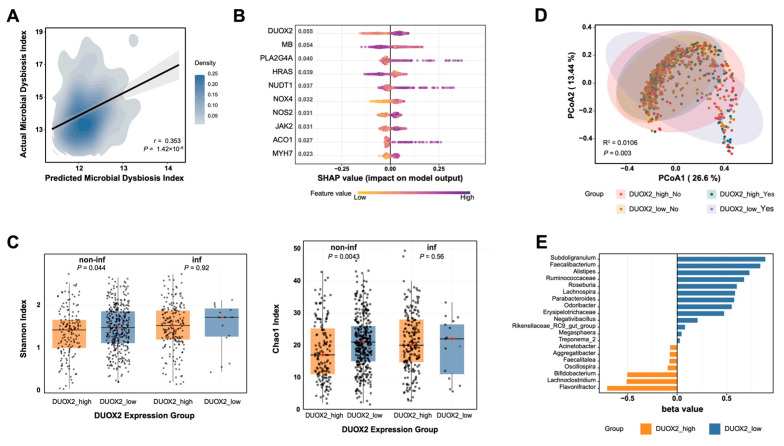
DUOX2 upregulation is associated with gut microbial dysbiosis in patients with CD: (**A**–**E**) Integrative analysis of gut microbial 16S rRNA sequencing data and host transcriptomes (RNA-Seq) from a large Dutch cohort of CD (*n* = 697). (**A**) Prediction of gut microbiota dysbiosis severity based on host gene expression using an extreme gradient boosting (XGBoost) model. (**B**) Visualization of the contribution of gene expression to microbiota dysbiosis using SHapley Additive exPlanations (SHAP) values. (**C**) α-Diversity (Chao1 and Shannon indices) among the *DUOX2* high-expression non-inflamed tissue group (*n* = 152), *DUOX2* low-expression non-inflamed tissue group (*n* = 334), *DUOX2* high-expression inflamed tissue group (*n* = 196), and *DUOX2* low-expression inflamed tissue group (*n* = 15). (**D**) PCoA of β-diversity based on Bray–Curtis metric distance among the above four groups. (**E**) LEfSe-generated bar plot showing genus-level bacterial taxa that differ significantly between the *DUOX2* high-expression group and the *DUOX2* low-expression group (*p* < 0.05). Blue bars indicate genera enriched in the *DUOX2* low-expression group, whereas orange bars indicate genera enriched in the *DUOX2* high-expression group.

To examine the relationship between intestinal epithelial OS and gut microbial ecology—specifically, whether DUOX2 is linked to the dysbiotic patterns characteristic of CD—mucosal transcriptomic data were integrated with paired 16S rRNA sequencing profiles from a large Dutch cohort. Machine learning-based modeling revealed that host gene expression profiles were strongly predictive of microbiota dysbiosis severity in patients with CD (*p* = 1.42 × 10^−9^), with *DUOX2* emerging as the most contributory OS-related gene (Figure 2A,B). Patients with high *DUOX2* expression exhibited significantly reduced microbial α-diversity, as reflected by lower Shannon and Chao1 indices, when biopsies were obtained from non-inflamed intestinal regions but not from inflamed regions (Figure 2C). However, β-diversity analysis demonstrated a distinct microbial community structure in the high-*DUOX2* groups associated with both non-inflamed and inflamed intestinal regions, indicating broad alterations in microbial communities in association with DUOX2 activation (Figure 2D).

At the genus level, elevated *DUOX2* expression was positively correlated with increased abundances of *Flavonitractor*, *Lachnoclostridium*, and *Bifidobacterium*, whereas low *DUOX2* expression was associated with enrichment of a distinct set of bacterial taxa, including *Subdoligranulum*, *Faecalibacterium*, *Alistipes*, members of the *Ruminococcaceae* family, and *Parabacteroides* (Figure 2E). Several taxa enriched in high-*DUOX2* mucosa have been reported as ROS-tolerant or ROS-inducible taxa [30,31], suggesting adaptation to a pro-oxidative intestinal environment. In contrast, *DUOX2* expression was negatively correlated with major short-chain fatty acid (SCFA)-producing microbial genera, including *Faecalibacterium* and *Subdoligranulum* [32], which are critical for maintaining colonic homeostasis. The depletion of beneficial commensals in high-*DUOX2* mucosa is aligned with a pro-oxidative, pro-inflammatory microbial milieu. Collectively, these findings demonstrate that DUOX2 upregulation is closely associated with gut microbial dysbiosis in CD, characterized by reduced microbial diversity, selective loss of oxygen-sensitive beneficial bacteria, and expansion of ROS-tolerant taxa. This DUOX2–microbiota axis supports a model in which epithelial OS contributes to ecological imbalances, potentially reinforcing intestinal inflammation.

### 3.3. Loss of DUOX2 Attenuates Intestinal Inflammation and Epithelial Oxidative Stress

**Figure 3:**
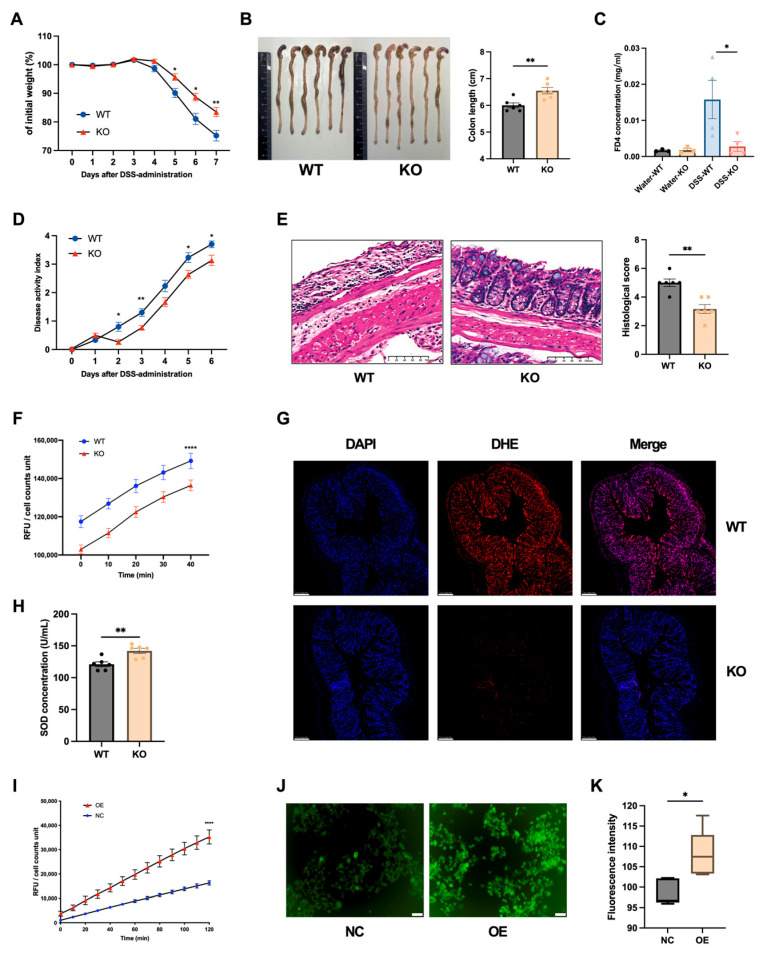
DUOX2 deficiency attenuates colitis inflammation and alters the oxidative stress: (**A**) Initial body weight and percentage weight change in *Duox2* KO and WT mice following 7 days of 2% DSS administration (*n* = 10 per group). (**B**) Colon length and colonic photographs from *Duox2* KO and WT mice at day 8 (*n* = 6 per group). (**C**) Serum levels of FD4 concentration in *Duox2* KO and WT mice at day 8 following administration of water and 2% DSS (*n* ≥ 3 per group). (**D**) Disease activity index (DAI) of *Duox2* KO and WT mice following 7 days of 2% DSS administration (*n* = 10 per group). (**E**) Representative high-magnification light microscopy images of H&E-stained colon tissue sections and histological inflammation scores from *Duox2* KO and WT mice at day 8 following 7 days of 2% DSS administration. Scale bars: 100 μm. Low-magnification panoramic images are provided in Figure S3. (**F**) H_2_O_2_ levels in colonic epithelial cells isolated from *Duox2* KO and WT mice at steady state (*n* = 6 per group). (**G**) Representative immunofluorescence images showing ROS levels in colonic tissues from *Duox2* KO and WT mice at steady state. Blue fluorescence indicates DAPI-labeled nuclei; red fluorescence indicates DHE-labeled ROS; merged images show the superimposition of the two fluorescence channels. Scale bars: 100 μm. (**H**) Serum SOD levels of *Duox2* KO and WT mice at steady state (*n* = 6 per group). (**I**) H_2_O_2_ levels in DUOX2-overexpressing NCM460 cells and control cells (*n* = 6 per group). (**J**) Representative immunofluorescence images showing ROS levels in DUOX2-overexpressing NCM460 cells and control cells (*n* = 5 per group). Green fluorescence indicates DCFH-DA-labeled ROS. (**K**) Fluorescence intensity of the cells shown in Panel J. Note: * *p* < 0.05, ** *p* < 0.01, **** *p* < 0.0001. Data are the representative of three independent experiments, and values represent means (±SEM).

To investigate the functional role of DUOX2 in intestinal inflammation, *Duox2* KO mice were analyzed. Quantitative PCR and Western blotting analyses confirmed the complete absence of DUOX2 in the colonic tissue of KO mice (Figure S2A,B). Under baseline conditions, no significant difference in body weight was observed between KO and WT mice (Figure S2C); however, KO mice exhibited a shorter colonic length than their WT counterparts (Figure S2D). Histological evaluation revealed no mucosal erosion or neutrophil infiltration in the colonic tissue of KO mice (Figure S2E). Additionally, the FD4 levels were comparable between KO and WT mice, and the colonic expression of the barrier-associated markers *Zo1* and *Occludin* also showed no significant differences (Figure 3C and Figure S2F). Furthermore, the expression of the inflammatory markers *Tnf*, *Il1β*, *Il6*, and *Lcn2* exhibited no statistically significant differences between KO and WT mice (Figure S2G), suggesting that DUOX2 knockout does not induce subclinical intestinal inflammation under baseline conditions. We next investigated the regulatory role of DUOX2 in intestinal inflammation. Following DSS administration, KO mice exhibited less body weight loss, lower disease activity, longer colonic lengths, and milder intestinal inflammation and mucosal injury, along with preserved intestinal barrier permeability, compared to WT controls (Figure 3A–E and Figure S3A). TNBS-induced colitis in KO mice produced similar protective effects (Figure S4A–D), indicating that the phenotype is independent of the colitis model.

Mucosal OS was assessed, as DUOX2 is a major epithelial source of H_2_O_2_. KO mice exhibited significantly lower H_2_O_2_ levels in intestinal epithelial cells, reduced DHE fluorescence intensity in colonic tissues, and elevated SOD levels compared with their WT controls (Figure 3F–H). Complementary in vitro experiments showed that DUOX2 overexpression in NCM460 intestinal epithelial cells increased ROS production, with a concurrent elevation in H_2_O_2_ levels and DCFH-DA fluorescence intensity (Figure 3I–K). These observations indicate that DUOX2 depletion is associated with substantially reduced OS.

### 3.4. DUOX2 Depletion Attenuates Colitis Through a Microbiota-Dependent Mechanism

**Figure 4:**
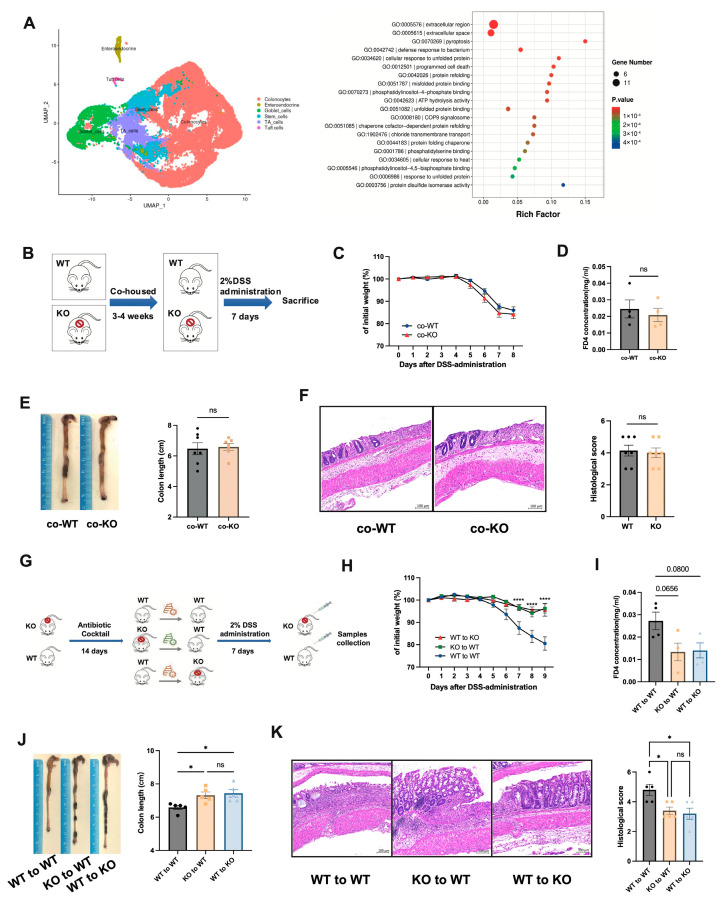
Microbiota from the *Duox2* KO mouse intestine suppresses colitis: (**A**) Single-cell RNA sequencing (scRNA-Seq) was performed on colonic epithelial cells from WT and *Duox2* KO mice (*n* = 2 per group). Differential gene expression analysis followed by Gene Ontology biological process enrichment showed significant enrichment of host–microbe interaction pathways. (**B**) Cohousing experiment design. *Duox2* KO and WT mice were cohoused (1:1 ratio) for 3–4 weeks to enable gut microbiota exchange. After cohousing, DSS-induced colitis was established by treating all mice with 2% (*w*/*v*) DSS in drinking water for 7 days. (**C**) Body weight of co-KO and co-WT mice following 7 days of 2% DSS treatment (*n* = 7 per group). (**D**) Serum levels of FD4 concentration from co-KO and co-WT mice at day 8 following 7 days of 2% DSS administration (*n* = 4 per group). (**E**) Colon length and colonic photographs from co-KO and co-WT mice at day 8 (*n* = 7 per group). (**F**) Representative high-magnification light microscopy images of H&E-stained colon tissue sections and histological inflammation scores from co-KO and co-WT mice at day 8 following 7 days of 2% DSS administration. Scale bars: 100 μm. Low-magnification panoramic images are provided in Figure S3. (**G**) FMT experimental design. D*uox2* KO and WT mice were put on a course of intragastrically antibiotic cocktail administration for 14 days for gut microbiota depletion prior to FMT and gavaged with the fecal contents of either WT or D*uox2* KO donor mice. After FMT, DSS-induced colitis was established. (**H**) Body weight of WT to WT, KO to WT, and WT to KO mice following 7 days of 2% DSS administration (*n* = 5 per group). (**I**) Serum levels of FD4 concentration from WT to WT, KO to WT, and WT to KO mice at day 9 following 7 days of 2% DSS administration (*n* = 4 per group). (**J**) Colon length and colonic photographs from WT to WT, KO to WT, and WT to KO mice at day 9 (*n* = 5 per group). (**K**) Representative high-magnification light microscopy images of H&E-stained colon tissue sections and histological inflammation scores from WT to WT, KO to WT, and WT to KO mice at day 9 following 7 days of 2% DSS administration. Scale bars: 100 μm. Low-magnification panoramic images are provided in Figure S3. Data were expressed as the mean ± SEM. Differences in the data were assessed by ordinary one-way ANOVA or the Kruskal–Wallis test, depending on the sample distribution. Note: * *p* < 0.05, **** *p* < 0.0001, ns: not significant.

Given the close association between DUOX2-mediated OS and gut microbial dysbiosis in CD, the potential microbiota-dependency of DUOX2 protection was examined; scRNA-Seq of colonic epithelial cells revealed that KO mice exhibited reduced activation of bacterial sensing, unfolded protein response, and misfolded protein binding pathways (Figure 4A), implicating a potential microbiota-driven mechanism underlying the observed protection.

Cohousing of KO and WT mice for 4 weeks prior to DSS treatment was used to normalize the microbiota composition (Figure 4B). After cohousing, the differential susceptibility to DSS-induced colitis between KO and WT mice was abolished, with no significant differences observed in body weight (Figure 4C), intestinal barrier permeability (Figure 4D), colon length (Figure 4E), and histological inflammation scores (Figure 4F and Figure S3), suggesting an association between the protective phenotype and gut microbial composition.

The FMT experiment was performed to further assess microbiota transmissibility (Figure 4G). Antibiotic-pretreated WT recipient mice receiving feces from *Duox2* KO donors exhibited attenuated disease severity, including reduced weight loss, improved intestinal barrier permeability, preserved colon length, and lower histological inflammation, compared with those receiving feces from WT donors (Figure 4H–K and Figure S3). Furthermore, *Duox2* KO mice receiving feces from WT donors also exhibited enhanced intrinsic resistance to DSS-induced intestinal injury compared with WT mice administered feces from WT donors, confirming that DUOX2 depletion is associated with a beneficial microbial community linked to resistance to colitis.

### 3.5. Parabacteroides Enrichment in Duox2 KO Mice Mediates Resistance to DSS-Induced Colitis

**Figure 5:**
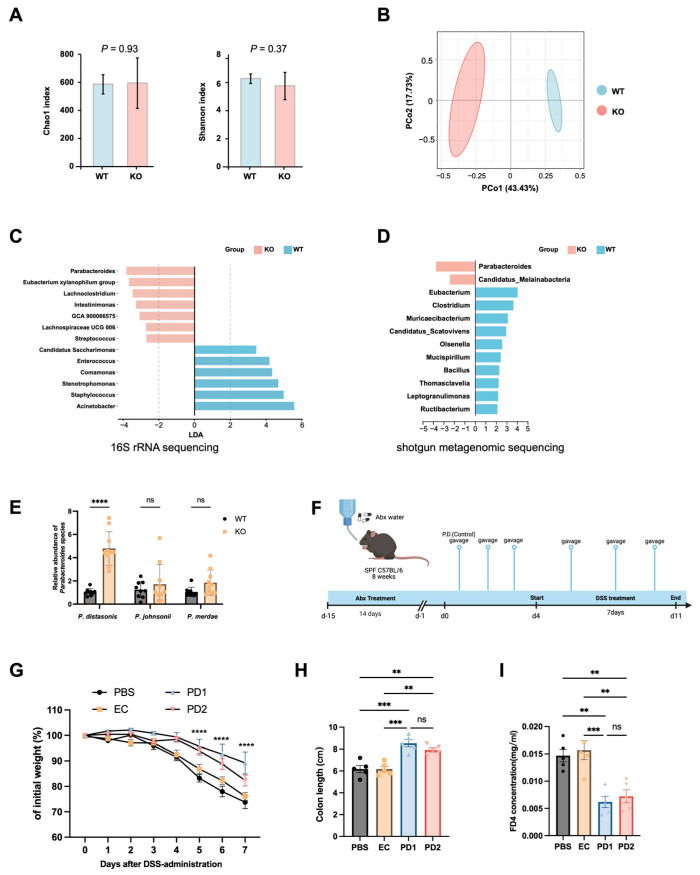
*Parabacteroides* enrichment in *Duox2* knockout mouse intestines alleviates DSS-colitis: (**A**–**C**) 16S rRNA sequencing was conducted on fecal bacterial DNA isolated from healthy *Duox2* KO and WT mice (*n* = 4 mice/group). (**A**) Alpha diversity profiles (Chao1 and Shannon indices). (**B**) PCoA of beta diversity based on Bray–Curtis dissimilarity. (**C**) LEfSe bar plot displaying genus-level taxonomic variations between *Duox2* KO and WT mice. Bacterial taxa with differential abundance were defined by an LDA score > 2. Blue bars represent genera enriched in WT mice, while pink bars indicate genera overrepresented in D*uox2* KO mice. (**D**) Metagenomic sequencing was performed on fecal bacterial DNA from healthy WT and *Duox2* KO mice (*n* = 5 mice/group). LEfSe bar plot illustrates genus-level taxonomic differences between the two groups (LDA > 2). Blue bars denote genera enriched in WT mice, and pink bars indicate genera overrepresented in *Duox2* KO mice. (**E**) qPCR was used to quantify the abundance of major *Parabacteroides* species in WT and *Duox2* KO mice (*n* = 10 mice/group). (**F**–**I**) *Parabacteroides* administration alleviated DSS-induced colitis. (**F**) Schematic of the *P. distasonis* intervention experiment. C57BL/6 WT mice received intragastric administration of an antibiotic cocktail for 14 days to deplete their gut microbiota. Three days prior to DSS exposure, mice were gavaged with either *P. distasonis* strain ATCC BAA-1295 (PD1; 1.0 × 10^9^ CFUs/200 μL sterile PBS per mouse), *P. distasonis* strain ATCC 8503 (PD2; 1.0 × 10^9^ CFUs per mouse), non-pathogenic *Escherichia coli* strain MG1655 (EC; 1.0 × 10^9^ CFUs per mouse, bacterial control), or sterile PBS. Following DSS administration, gavage was continued every other day. (**G**) Body weight changes in the PD1, PD2, EC, and PBS groups (*n* = 5 mice/group). (**H**) Colon length measurements across the four groups (*n* = 5 mice/group). (**I**) Serum FD4 concentrations in the four groups. Data are presented as the mean ± SEM. Statistical analyses were performed using ordinary one-way ANOVA or the Kruskal–Wallis test, depending on data distribution. Note: ** *p* < 0.01, *** *p* < 0.001, **** *p* < 0.0001, ns: not significant.

To investigate potential microbiota-related differences, the gut microbial communities of *Duox2* KO and WT mice were profiled prior to DSS exposure; 16S rRNA sequencing revealed comparable α-diversity between groups, as assessed by the Chao1 and Shannon indices (Figure 5A). In contrast, β-diversity analysis based on Bray–Curtis and unweighted UniFrac distances demonstrated clear separation between *Duox2* KO and WT mice (Figure 5B and Figure S5A), indicating a substantial restructuring of community composition rather than a simple loss or gain of diversity. LEfSe analysis showed that the relative abundances of *Parabacteroides*, *Eubacterium xylanophilum*, and members of the *Lachnospiraceae* family were significantly increased in *Duox2* KO mice, whereas WT mice harbored higher abundances of opportunistic pathogens, including *Acinetobacter*, *Staphylococcus*, and *Stenotrophomonas* (Figure 5C).

Shotgun metagenomic sequencing yielded consistent results, with *Parabacteroides* identified as the most enriched genus in D*uox2* KO mice (Figure S5B,C). LEfSe analysis of metagenomic data further confirmed the enrichment of *Parabacteroides* in *Duox2* KO mice (Figure 5D). These results strongly point to *Parabacteroides* as a candidate effector genus responsible for the microbiota-mediated protection observed in *Duox2* deficient mice.

*Parabacteroides* is increasingly recognized as a beneficial taxon with immunomodulatory properties, inflammation-attenuating capacity, and roles in metabolic support across multiple physiological contexts [33,34]. We therefore quantified the abundance of four major *Parabacteroides* species in *the Duox2* KO and WT groups via real-time qPCR with species-specific primers (Figure 5E). Among these, *Parabacteroides goldsteinii* was below the limit of detection, whereas *Parabacteroides distasonis* (*P. distasonis*) exhibited the most pronounced increase (Figure 5E).

To functionally test whether *P. distasonis* mediates the protective phenotype, antibiotic-pretreated WT mice were orally gavaged once daily with *P. distasonis* strain ATCC BAA-1295 (PD1; 1.0 × 10^9^ CFUs per mouse), *P. distasonis* strain ATCC 8503 (PD2; 1.0 × 10^9^ CFUs per mouse), non-pathogenic *E. coli* strain MG1655 (EC; 1.0 × 10^9^ CFUs per mouse, serving as a bacterial control), or PBS for 3 days before DSS administration (Figure 5F). Remarkably, both *P. distasonis* strains significantly ameliorated DSS-induced colitis, as evidenced by reduced weight loss, preserved colon length, and improved intestinal barrier integrity (Figure 5G–I).

Collectively, these results highlight *P. distasonis* as a leading DUOX2-associated effector microbe. Accordingly, we employed *P. distasonis* in the subsequent experiments to dissect the mechanisms by which DUOX2-driven OS shapes gut microbial composition and influences colitis outcomes.

### 3.6. DUOX2-Dependent Oxidative Stress Restricts Parabacteroides Proliferation

**Figure 6:**
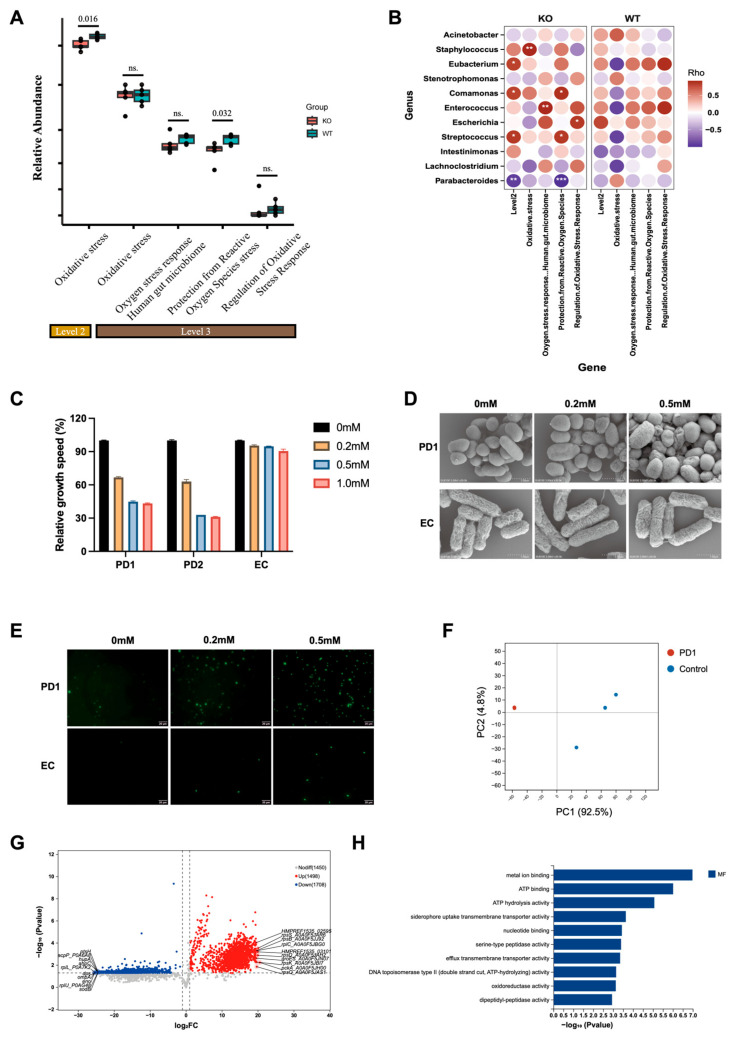
DUOX2-dependent oxidative stress restricts *Parabacteroides* proliferation: (**A**) Functional profiling of OS based on gut metagenomic data from *Duox2* KO and WT mice. (**B**) Correlation analysis between differential bacterial genera and OS profiling in *Duox2* KO and WT mice. Color key: Spearman’s correlation coefficient (Rho). Red gradients indicate positive correlations, purple gradients indicate negative correlations, and white indicates no correlation. (**C**) Relative growth of *P. distasonis* strains and *E. coli* under different hydrogen peroxide (H_2_O_2_) concentrations. (**D**) Scanning electron microscopy (SEM) images showing morphological changes in *P. distasonis* and *E. coli* under different H_2_O_2_ concentrations. (**E**) Representative immunofluorescence images showing ROS levels in *P. distasonis* and *E. coli* under different H_2_O_2_ concentrations. (**F**–**H**) Proteomics sequencing analysis of *P. distasonis* and *E. coli*, with three biological replicates for each strain. (**F**) PCA of *P. distasonis* and *E. coli*, presenting the first and second principal components. (**G**) Volcano plot depicting the identified proteins in the two species. The significantly differentially abundant proteins are shown as blue dots and red dots, representing downregulated and upregulated proteins, respectively. The nonsignificant proteins are shown in gray. The screening criteria were *p* < 0.05 and |FC| ≥ 2. (**H**) Gene Ontology pathway analysis of differentially expressed proteins at the molecular function (MF) level (*p* < 0.05). Exact *p* levels were all provided. PD1, *P. distasonis* strain ATCC BAA-1295; PD2, *P. distasonis* strain ATCC 8503; EC, *E. coli* MG1655. Note: * *p* < 0.05, ** *p* < 0.01, *** *p* < 0.001, ns: not significant.

To assess whether DUOX2-mediated redox stress directly influences the growth of *P. distasonis*, the microbial OS-response capacity was analyzed in *Duox2* KO mice, which exhibited a significant reduction in the abundance of genes involved in global OS responses, suggesting a less oxidizing luminal environment in the absence of DUOX2 (Figure 6A). Correlation analysis further revealed that, among all taxa enriched in *Duox2* KO mice, *Parabacteroides* showed a strong negative association with OS-response gene abundance (*p* < 0.01) (Figure 6B), indicating that DUOX2-dependent OS is linked to the restriction of *Parabacteroides* expansion within the gut ecosystem.

To directly test this relationship, in vitro H_2_O_2_ challenge assays were performed. Minimal medium was supplemented with increasing H_2_O_2_ concentrations (0, 0.2, 0.5, or 1.0 mM) to mimic the localized DUOX2-derived H_2_O_2_ microenvironment. In contrast to *E. coli* strain EC, both *P. distasonis* strains (PD1 and PD2) were highly sensitive to H_2_O_2_. At 1.0 mM H_2_O_2_, neither PD1 nor PD2 exhibited growth beyond their initial OD_600_ (0.1), and even at 0.2 mM H_2_O_2_, growth was reduced by more than 30% (Figure 6C and Figure S6). Scanning electron microscopy revealed morphological alterations under increasing H_2_O_2_ exposure (Figure 6D). PD1 cells displayed extensive membrane disruption and widespread lysis with rising H_2_O_2_ concentrations, whereas EC cells exhibited only modest morphological damage. Intracellular ROS levels increased sharply under H_2_O_2_ treatment and remained substantially higher than in the EC strain (Figure 6E). These findings indicate that *P. distasonis* lacks sufficient intrinsic OS-buffering capacity, making it highly susceptible to DUOX2-generated H_2_O_2_.

Label-free quantitative proteomics was conducted to investigate the molecular basis of this differential tolerance. PCA of proteomics data revealed distinct proteomic profiles between PD1 and EC groups (*n* = 3 per group) (Figure 6F). Volcano plot analysis using cutoffs of *p* < 0.05 and fold change ≥ 2 identified 3208 differentially expressed proteins (DEPs), including 1498 upregulated and 1708 downregulated in PD1 relative to EC (Figure 6G). Notably, several key redox-detoxifying enzymes (SodB and AhpC) were among the most downregulated proteins in PD1. AhpC serves as a primary H_2_O_2_ scavenger in many gut commensals, and its deficiency provides a compelling mechanistic basis for the pronounced H_2_O_2_ sensitivity of *P. distasonis*. GO enrichment further indicated that DEPs were significantly associated with oxidoreductase activity and other OS-related molecular functions (*p* < 0.05) (Figure 6H), reinforcing the notion that intrinsic redox-detoxification limitations underlie the vulnerability of *Parabacteroides* to DUOX2-derived OS.

### 3.7. DUOX2 Inhibition Mitigates DSS Colitis While Reducing Oxidative Stress and Restoring Microbial Balance

**Figure 7:**
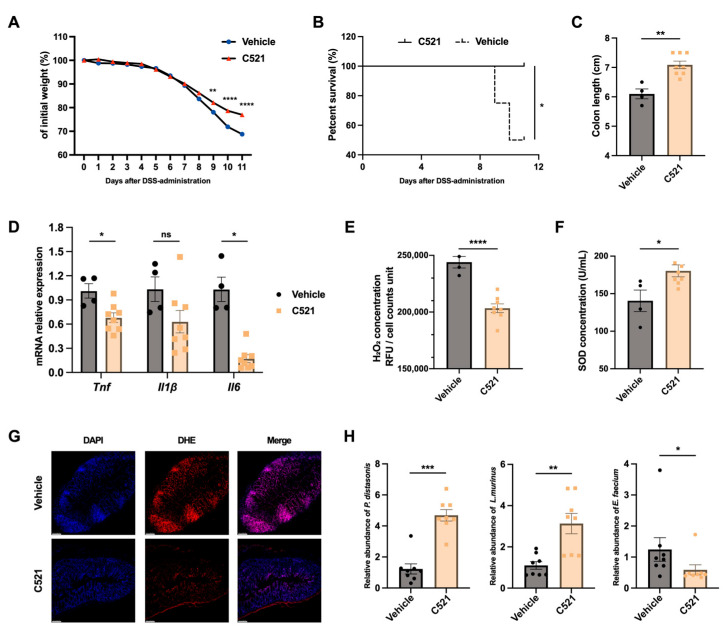
The DUOX2 inhibitor Compound 521 alleviates DSS-induced colitis, reduces oxidative stress, and restores gut dysbiosis in mice: (**A**) Body weight changes in mice treated with Compound 521 (20 ng/kg) or vehicle via daily intraperitoneal (IP) injection following DSS treatment (*n* = 8 per group). (**B**) Survival rates of mice treated with Compound 521 (20 ng/kg) or vehicle via daily IP injection following DSS treatment. The data are presented as Kaplan–Meier survival curves (*n* = 8 per group). (**C**) Colon lengths of mice treated with Compound 521 (20 ng/kg) or vehicle via daily IP injection following DSS treatment (*n* ≥ 4 per group). (**D**) Quantitative PCR analysis of inflammatory cytokine mRNA levels (*Tnf*, *Il1β*, and *Il6*) in mice treated with Compound 521 (20 ng/kg) or vehicle via daily IP injection following DSS treatment (*n* ≥ 4 per group). (**E**) H_2_O_2_ levels in colonic epithelial cells isolated from mice treated with Compound 521 (20 ng/kg) or vehicle via daily IP injection following DSS treatment (*n* ≥ 4 per group). (**F**) Serum SOD levels of mice treated with Compound 521 (20 ng/kg) or vehicle via daily IP injection following DSS treatment (*n* ≥ 4 per group). (**G**) Representative immunofluorescence images showing ROS levels in colonic tissues from Compound 521- or vehicle-treated mice following DSS treatment. Blue fluorescence indicates DAPI-labeled nuclei; red fluorescence indicates DHE-labeled ROS; merged images show the superimposition of the two fluorescence channels. (**H**) Quantitative PCR analysis of fecal microbial abundance (*Parabacteroides distasonis*, *Lactobacillus murinus*, and *Enterococcus faecium*) in mice treated with Compound 521 (20 ng/kg) or vehicle via daily IP injection following DSS treatment (*n* = 8 per group). Note: * *p* < 0.05, ** *p* < 0.01, *** *p* < 0.001, **** *p* < 0.0001, ns: not significant. The data are representative of three independent experiments, and the error bars indicate the means ± SEMs. C521, Compound 521.

To pharmacologically validate the role of DUOX2-mediated OS in the pathogenesis of colitis, a reported selective DUOX2 inhibitor, Compound 521 [35,36], was tested in the DSS-induced colitis model. Mice received 20 ng/kg/day via intraperitoneal injection following DSS administration. By day 9, Compound 521-treated mice exhibited significantly less body weight loss, improved survival, and longer colon lengths compared with vehicle controls (Figure 7A–C). Colonic expression of the inflammatory markers *Il1β*, *Il6*, and *Tnf* was also significantly lower in treated mice (Figure 7D).

Given the central role of DUOX2 in epithelial ROS generation, we examined OS status following pharmacological inhibition. DUOX2 blockade effectively reduced OS, as evidenced by decreased H_2_O_2_ concentrations, reduced DHE fluorescence, and increased SOD activity (Figure 7F,G). The intestinal microbiota was also affected, with higher abundances of *P. distasonis* and other beneficial taxa (e.g., *Lactobacillus murinus*) and reduced *Enterococcus faecium* in treated mice (Figure 7H).

Collectively, these findings demonstrate that pharmacological DUOX2 inhibition reduces OS, partially restores microbial balance, and mitigates DSS-induced colitis. These data are consistent with genetic loss-of-function and microbial transfer studies, supporting a mechanistic model in which epithelial DUOX2 generates a pro-oxidative luminal environment that destabilizes microbial homeostasis and amplifies intestinal inflammation.

## 4. Discussion

CD is characterized by chronic intestinal inflammation driven by dysregulated host–microbiome interactions and intestinal immune dysregulation, with OS increasingly recognized as an upstream contributor to epithelial intestinal barrier disruption and microbial imbalance [2,4,37]. DUOX2 is consistently upregulated in patients with CD and represents a major epithelial source of ROS. However, whether DUOX2-derived OS actively shapes gut microbial ecology—and thereby contributes to CD progression—remains unclear. In this study, by integrating human multi-omics analyses, IEC-specific *Duox2* knockout mice, in vitro bacterial redox assays, and pharmacological intervention, we address this knowledge gap and propose a mechanistic framework in which epithelial DUOX2 drives a redox-dependent shift in microbial ecology that amplifies intestinal inflammation.

Our findings extend prior observations identifying *DUOX2* as one of the most strongly induced OS-related genes in CD mucosa. Although genome-wide association studies (GWAS) have not classified *DUOX2* as a canonical CD risk gene, accumulating evidence links *DUOX2* to IBD susceptibility [38,39,40]. Our previous large-scale transcriptomic meta-analysis further underscored *DUOX2* as one of the most prominently upregulated OS-related genes in CD intestinal tissues [7]. Building on this, the current study integrates transcriptomic and microbial sequencing data to validate a more consistent association between elevated *DUOX2* expression and microbial dysbiosis. Specifically, high-*DUOX2* individuals exhibited an altered microbial composition, with depletion of beneficial anaerobes and enrichment of ROS-tolerant pathobionts. In addition, high-*DUOX2* individuals exhibited upregulation of pro-inflammatory pathways. Given the well-established bidirectional relationship between intestinal inflammation and microbiota homeostasis, these findings underscore the ecological relevance of DUOX2-mediated redox imbalance in CD pathogenesis [41]. Notably, bioinformatics modeling further demonstrated that DUOX2 expression could reliably predict the degree of microbial dysbiosis in patients with CD, supporting the notion that DUOX2 functions as an upstream determinant of mucosal redox balance with broad ecological and immunological consequences, rather than merely reflecting inflammatory status. Nevertheless, it is critical to acknowledge that these observations reflect correlative associations derived from cross-sectional transcriptomics data; as such, a definitive causal relationship between *DUOX2* expression and microbial dysbiosis in human CD cannot be established, and the directionality of this interaction remains strictly inferential at this stage.

To further delineate the functional role of DUOX2 in intestinal homeostasis and disease susceptibility, we generated an IEC-specific *Duox2* KO mouse model. Phenotypic analyses revealed that DUOX2 ablation conferred marked protection against experimental colitis and altered the intestinal OS profile. These loss-of-function findings complement and extend previous gain-of-function studies in TLR4-driven DUOX2 transgenic mice, in which DUOX2 overexpression exacerbated epithelial barrier dysfunction and subclinical inflammation [11]. Together, these reciprocal genetic approaches support a causal role for DUOX2 in promoting intestinal inflammation through the disruption of redox homeostasis and epithelial barrier integrity.

Importantly, the cohousing and FMT experiments demonstrated that the protective phenotype associated with DUOX2 deletion is microbiota-dependent and transmissible. These findings establish a functional pathway in which epithelial DUOX2 activity shapes gut microbial composition, thereby modulating host susceptibility to inflammation. When combined with epithelial single-cell transcriptomics data indicating reduced stress response and pathogen-sensing signatures, these results position DUOX2 as a key architect of the intestinal microbial ecosystem.

Multi-omics analyses across mouse and human datasets converged on *Parabacteroides*, particularly *P. distasonis*, as a DUOX2-sensitive effector taxon. Depletion of *Parabacteroides* was consistently observed in high-DUOX2 patients, WT mice, and DUOX2-overexpressing contexts, whereas enrichment was evident in DUOX2-deficient mice and low-DUOX2 human samples. Functional validation demonstrated that oral administration of *P. distasonis* significantly ameliorated DSS-induced colitis and improved epithelial barrier integrity, supporting its role as a protective commensal that is selectively disadvantaged under DUOX2-driven oxidative conditions. This cross-species convergence identifies *P. distasonis* as a key microbial mediator of DUOX2-associated ecological imbalance.

*Parabacteroides* is a core taxon of the human gut microbiota and has been widely reported to exert beneficial effects, including modulation of mucosal immunity, attenuation of inflammation, and support of carbohydrate metabolism, leading to its recognition as a promising probiotic candidate [33,34,42]. Previous studies have reported reduced *Parabacteroides* abundance in patients with IBD, with *P. distasonis* showing particularly pronounced depletion in inflamed mucosal tissues [33,43]. Our findings extend these findings by demonstrating that *P. distasonis* supplementation can mitigate intestinal inflammation and restore epithelial barrier function, reinforcing its therapeutic relevance in IBD contexts.

Mechanistically, our study pursued two complementary lines of investigation: first, metagenomic analyses revealed that DUOX2 ablation was associated with altered microbial OS-response profiles, with *Parabacteroides* consistently linked to lower microbial OS signatures. These data suggest that DUOX2-driven redox conditions shape the intestinal OS microenvironment in a manner that is unfavorable for *Parabacteroides* persistence. Second, in vitro experiments directly validated this relationship, showing that elevated H_2_O_2_ concentrations markedly inhibited *P. distasonis* growth, induced morphological damage, and increased intracellular ROS accumulation. Proteomic profiling further elucidated the molecular basis of this sensitivity: unlike H_2_O_2_-tolerant *E. coli*, *P. distasonis* lacked key antioxidant enzymes, particularly AhpC and SodB. AhpC is well established as a primary H_2_O_2_ scavenger in many gut commensals [44,45], and its absence has been shown to result in rapid ROS accumulation and cellular damage in bacterial systems [46]. The deficiency of these detoxification pathways in *P. distasonis* provides a plausible mechanistic explanation for its selective depletion under DUOX2-mediated oxidative conditions.

While the downstream protective mechanisms provided by *P. distasonis* to the host are not the primary focus of this study, we have obtained preliminary data supporting potential functional pathways. Specifically, both in vitro and in vivo experiments confirmed that *P. distasonis* exerts a colitis-alleviating effect by restoring mucosal barrier function, which provides preliminary validation of its host-associated protective mechanisms. Additionally, metabolomic analysis revealed that *P. distasonis* may further regulate mucosal barrier integrity and influence colitis progression by modulating bile acid metabolism, proline metabolism, and other relevant pathways. Future studies will aim to delineate the precise molecular cascades underlying these metabolic and barrier-modulating effects, as well as their functional crosstalk, in order to fully exploit the therapeutic potential of *P. distasonis* in CD.

Finally, pharmacological inhibition of DUOX2 using Compound 521 recapitulated key features of genetic DUOX2 deletion, including reduced epithelial ROS levels, attenuation of colitis severity, and partial restoration of beneficial microbial taxa such as *P. distasonis*. These results nonetheless highlight the therapeutic potential of targeting DUOX2 to modulate mucosal redox balance and restore gut microbial homeostasis, with the caveat that potential off-target effects and NOX-family cross-reactivity of Compound 521 were not experimentally validated in our in vivo studies. Beyond DUOX2 inhibition, our findings also suggest the possibility of leveraging ROS-sensitive commensals such as *P. distasonis* as next-generation probiotics for the management of intestinal inflammation. Given the intrinsic sensitivity of *P. distasonis* to ROS, restoring a normoxidative intestinal niche is an essential prerequisite for its colonization and subsequent therapeutic efficacy in colitis. A rational sequential therapeutic strategy is therefore proposed, wherein patients first receive conventional anti-inflammatory interventions (e.g., anti-TNF-α therapy) or DUOX2-targeted therapy to alleviate intestinal oxidative inflammation and normalize the microenvironment, followed by *P. distasonis* supplementation to further rebalance the dysregulated gut microbiota. Notably, treatment with anti-TNF-α, DUOX2 inhibition, and *P. distasonis* supplementation may be clinically relevant, especially in patients who develop resistance to anti-TNF-α therapy. This therapeutic paradigm merits further investigation.

Collectively, this study reveals a previously unrecognized mechanism by which epithelial DUOX2 shapes gut microbial ecology through redox-dependent niche selection, thereby exacerbating intestinal inflammation. Mechanistically, it establishes DUOX2 as a proximal regulator of gut homeostasis acting through microbial ecological remodeling, rather than exclusively through immune-mediated pathways. Therapeutically, it supports DUOX2 inhibition and restoration of ROS-sensitive beneficial taxa (e.g., *P. distasonis*) as potential strategies for the treatment of CD. Conceptually, it underscores the importance of epithelial-derived ROS as a selective ecological force governing microbial community structure.

This study has several limitations. First, although *P. distasonis* emerged as a dominant DUOX2-sensitive effector taxon, the roles of other *Parabacteroides* species warrant further investigation. Second, while we identified the absence of AhpC and SodB as a key contributor to *P. distasonis* H_2_O_2_ sensitivity, the downstream mechanisms underlying this bacterial growth arrest were not fully elucidated; targeted genetic manipulation of these antioxidant pathways would provide valuable mechanistic insight. Finally, although previous studies have shown that microbiota can induce DUOX2 expression, our work demonstrates the reciprocal effect of DUOX2 on microbiota composition. Dissecting the directionality and feedback dynamics of this bidirectional crosstalk in CD remains an important challenge for future research.

In conclusion, this study establishes a mechanistic link between the epithelial DUOX2–ROS–microbiota axis and colitis susceptibility. Integrating human and mouse multi-omics data with mechanistic functional analyses, we identified DUOX2 as a key epithelial regulator of redox-driven microbial ecology and intestinal inflammation. Therefore, targeting DUOX2 or leveraging ROS-sensitive beneficial microbes represents a promising, mechanism-based therapeutic strategy for CD.

## Data Availability

The datasets generated and/or analyzed during the current study are available from a combination of public repositories and the corresponding authors, as detailed below. Publicly available datasets include intestinal RNA-Seq and mucosal 16S rRNA sequencing data from patients with Crohn’s disease in a Dutch cohort, which are deposited in the European Genome-Phenome Archive (EGA) under accession code EGAS00001002702 (mucosal RNA-Seq: EGAD00001008214; 16S rRNA sequencing: EGAD00001008215). Human single-cell RNA-Seq datasets were obtained from the Single Cell Portal (https://singlecell.broadinstitute.org/single_cell) (accessed on 22 July 2025), including data from the PREDICT 2021 studies (CD and FGID), which are available in the public domain. Due to ethical and privacy restrictions, raw intestinal RNA-Seq data from the FAH–SYS cohort, murine intestinal microbiota sequencing data, murine single-cell RNA-Seq data, and bacterial proteomics datasets are not publicly available but can be obtained from the corresponding authors upon reasonable request and subject to approval by the relevant ethics committees.

## Supplementary Materials

The following supporting information can be downloaded at https://www.mdpi.com/article/10.3390/antiox15030292/s1: Figure S1: DUOX2 expression correlates with intestinal inflammation in mice with colitis; Figure S2: DUOX2 deficiency does not induce overt intestinal pathology under steady-state conditions; Figure S3: Low-magnification H&E staining of colon tissues from DSS-treated, co-housed and FMT-treated *Duox2*-KO/WT mice; Figure S4: *Duox2* knockout mice are refractory to TNBS-induced colitis; Figure S5: *Duox2* deficiency alters the gut microbiota; Figure S6: *Parabacteroides distasonis* is sensitive to endogenous oxidative stress. Table S1: Demographic and clinical characteristics of patients with CD and normal controls; Table S2: List of primers used in this study; Table S3: Expression profiles of NADPH oxidase family members in the FAH-SYS cohort based on transcriptomic data.
