# Supplementary material for: DUOX2-Driven Oxidative Stress Alters the Gut Redox Niche and Promotes Microbial Dysbiosis in Crohn’s Disease
Source: Antioxidants (Basel). 2026 Feb 26;15(3):292. doi: 10.3390/antiox15030292 (PMC13023900; doi:10.3390/antiox15030292)
Supplement: Supplementary file 1 [file antioxidants-15-00292-s001.zip › antioxidants-4078938-supplementary1/Supplementary files/Supplementary figure legends_revised.docx]

**Supplementary figure legends**

**Figure S1. DUOX2 expression correlates with intestinal inflammation in mice with colitis**. (**A**) qPCR results of relative DUOX2 expression in colonic tissues from DSS-induced colitis models at different stages: Day 0 (n = 10), Day 4 (early inflammatory phase, n = 10), Day 9 (acute phase, n = 10), and Day 20 (recovery phase, n = 8). (**B**) qPCR results of relative DUOX2 expression in colonic tissues from TNBS-induced colitis models: TNBS-induced group (n = 5) and control group (n = 5). (**C**) Representative DUOX2 protein levels in colonic tissues from DSS-induced colitis detected by Western blotting: Lines 1–3 represent the non-model group (Day 0); lines 4–6 represent the early inflammatory phase (Day 4); lines 7–9 represent the acute inflammatory phase (Day 9); and lines 10–12 represent the recovery phase (Day 20), respectively. (**D**) Immunohistochemical staining of DUOX2 in colonic tissues from DSS-induced colitis models at different stages. **p* < 0.05, ***p* < 0.01, ****p* < 0.001, *****p* < 0.0001.

**Figure S2. DUOX2 deficiency does not induce overt intestinal pathology** **under steady-state conditions.** (**A**) qPCR results of relative *Duox2* expression in colonic tissues from *Duox2*-KO and WT mice (n = 3 per group). (**B**) Representative DUOX2 protein levels in colonic tissues from *Duox2*-KO and WT mice detected by Western blotting (n = 3 per group). (**C**) Body weight of *Duox2*-KO and WT mice from 3-11 weeks of age (n = 6 per group). (**D**) Colon length and representative photographs of colons from *Duox2*-KO and WT mice at 8 weeks of age (n = 6 per group). **(E)** Representative light microscopy images of H&E-stained colonic tissue sections from Duox2-KO and WT mice at 8 weeks of age. Scale bars: 1000μm; (**F**) qPCR analysis of barrier-associated marker expression in colonic tissues from *Duox2*-KO and WT mice (n = 3 per group). (**G**) qPCR analysis of inflammation markers expression in colonic tissues from *Duox2*-KO and WT mice (n = 3 per group). Scale bars: 1000μm. **p* < 0.05, ****p* < 0.001.

**Figure S3.** **Low-magnification H&E staining of colon tissues from DSS-treated, co-housed and FMT-treated *Duox2*-KO/WT mice. (A)** Representative low-magnification light microscopy images of H&E-stained colon tissue sections from *Duox2* KO and WT mice at day 8 following 7 days of 2% DSS administration. **(B)** Representative low-magnification light microscopy images of H&E-stained colon tissue sections and histological inflammation scores from co-KO and co-WT mice at day 8 following 7 days of 2% DSS administration. Scale bars: 1000 μm. **(C)** Representative low-magnification light microscopy images of H&E-stained colon tissue sections and histological inflammation scores from WT to WT, KO to WT, and WT to KO mice at day 9 following 7 days of 2% DSS administration. Scale bars: 1000 μm. Note: The corresponding high-magnification images are shown in Figure 3E, 4F, and 4K.

**Figure S4. *Duox2* knockout mice are refractory to TNBS-induced colitis.** (**A**) Initial weight and percent body weight in *Duox2*-KO and WT mice after TNBS treatment (n = 5 per group). (**B**) Representative photographs of colonic tissues from *Duox2*-KO and WT mice following TNBS induction. (**C**) Colon length and photographs of colons from *Duox2*-KO and WT mice after TNBS administration (n = 5 per group). (**D**) Representative light microscopy images of H&E-stained colonic tissue sections from *Duox2*-KO and WT mice after TNBS administration. Scale bars: 1000μm. ***p* < 0.01, ****p* < 0.001, *****p* < 0.0001.

**Figure S5. *Duox2* deficiency alters the gut microbiota.** (**A**) Principal coordinate analysis (PCoA) of beta diversity based on the Weighted UniFrac distance from 16S rRNA gene sequencing of fecal bacterial DNA in WT and *Duox2*-KO littermates. (**B****–C**) Metagenomic sequencing analysis was performed on fecal bacterial DNA from healthy WT and *Duox2*-KO littermates (n=5 per group). (**B**) Alpha diversity (assessed by Chao1 and Shannon indices). (**C**) PCoA of beta diversity based on the Jaccard distance.

**Figure S6.** ***Parabacteroides* *distasonis* is sensitive to endogenous oxidative stress.** The figure shows the growth curves of P*. distasonis* strain PD1 and *E. coli* at different hydrogen peroxide (H_2_O_2_) concentrations. (**A**) 0nM; (**B**) 0.2mM; (**C**) 0.5mM; (**D**) 1.0mM.
